# Food insecurity and its associations with cardiometabolic health in Latino individuals of Mexican ancestry

**DOI:** 10.3389/fnut.2024.1499504

**Published:** 2024-10-09

**Authors:** Ludovica Verde, Lindsay N. Kohler, Giovanna Muscogiuri, Oscar Parra, Yann C. Klimentidis, Dawn K. Coletta, Lawrence J. Mandarino

**Affiliations:** ^1^Department of Public Health, University of Naples Federico II, Naples, Italy; ^2^Unità di Endocrinologia, Diabetologia e Andrologia, Dipartimento di Medicina Clinica e Chirurgia, Centro Italiano per la Cura e il Benessere del Paziente con Obesità (C.I.B.O), Università Degli Studi di Napoli Federico II, Naples, Italy; ^3^Center for Disparities in Diabetes, Obesity, and Metabolism, University of Arizona Health Sciences, University of Arizona, Tucson, AZ, United States; ^4^Pima County Health Department, Epidemiology Division, Tucson, AZ, United States; ^5^Dipartimento di Medicina Clinica e Chirurgia, Unità di Endocrinologia, Diabetologia e Andrologia, Università Degli Studi Di Napoli Federico II, Naples, Italy; ^6^Cattedra Unesco “Educazione Alla Salute e Allo Sviluppo Sostenibile”, University Federico II, Naples, Italy; ^7^Department of Medicine, Division of Endocrinology, University of Arizona, Tucson, AZ, United States

**Keywords:** food insecurity, Latino/Hispanic, diet, type 2 diabetes, glycemic control

## Abstract

**Background:**

Latino populations, especially those of Mexican ancestry, face higher rates of both cardiometabolic diseases and food insecurity, compounding negative health outcomes. Food insecurity is associated with poor dietary choices, which not only worsen cardiometabolic health but also contribute to various health complications, making it a critical public health issue in these communities. The aim of this study was to investigate the prevalence of food insecurity and its associations with diet, cardiometabolic risk factors, and glycemic control among Latino individuals of Mexican ancestry.

**Methods:**

Cross-sectional observational study using data from the El Banco por Salud biobank. The study included 1,827 participants with a mean age of 52.5 ± 14.4 years, recruited from community-based settings. The majority were women (67.6%), obese (mean BMI 32.4 ± 7.0 kg/m^2^), and sedentary (43.5%). Food insecurity was assessed using the 6-item U.S. Household Food Security Module, while dietary information was obtained using the Brief Dietary Assessment Tool for Hispanics. Primary outcomes included cardiometabolic risk factors and glycemic control, specifically HbA1c levels.

**Results:**

Of 1,827 participants, 69.7% were food secure and 30.3% were food insecure. Food insecure participants had a significantly larger waist circumference (*p* = 0.034), consumed poorer quality diets, and had higher HbA1c levels (*p* = 0.043), with the association remaining significant after adjustments (*p* = 0.007 for age, sex, BMI, and waist circumference; *p* = 0.021 for additional sociodemographic factors).

**Conclusion:**

The findings reveal that food insecurity among Latino individuals of Mexican ancestry is associated with unhealthy food choices and higher HbA1c levels, exacerbating the risk of poor cardiometabolic health. This underscores an urgent need for targeted interventions to address food insecurity, ultimately promoting better metabolic health outcomes in vulnerable populations.

## 1 Introduction

Food insecurity, as defined by the Food and Agriculture Organization (FAO), occurs when “all people, at all times, lack physical and economic access to sufficient, safe, and nutritious food to meet their dietary needs and food preferences for an active and healthy life” (1). This broad definition encompasses four key dimensions: availability, access, utilization, and stability. To comprehensively evaluate the various dimensions of food insecurity, multiple assessment tools have been developed over the years (2). Two of the most widely recognized and validated instruments are the USDA Household Food Security Survey Module (HFSSM) and the Food Insecurity Experience Scale (FIES). These tools capture food insecurity at both the household and individual levels, making them suitable for a range of populations (2).

Food insecurity disproportionately affects low-income and minority populations in the United States (3, 4). In 2022, 12.8% of all U.S. households were classified as food insecure, with the prevalence rising to 20.8% among Hispanic households (3). Among Mexican immigrants and Hispanic farmworkers in the U.S., food insecurity rates are alarmingly high, ranging from 30 to 80% (4–7). Additionally, data from the 1999–2002 National Health and Nutrition Examination Surveys (NHANES) revealed that type 2 diabetes (T2D) prevalence was higher among U.S. individuals experiencing severe food insecurity than in people who reported being food secure, even after adjusting for body mass index (BMI) (8).

Food insecurity is strongly associated with poor health outcomes, including an increased risk of obesity and sarcopenic obesity, T2D, and cardiovascular disease (9, 10). Of note, sarcopenic obesity, characterized by the coexistence of excess adiposity and loss of muscle mass, presents a significant cardiometabolic risk (11), especially among food-insecure individuals, who tend to have lower diet quality and engage in sedentary behavior (12). Among individuals with T2D, food insecurity is linked to worse glycemic control, as defined by hemoglobin A1c (HbA1c) (13). One of the key mechanisms driving this relationship is poor diet quality, which is often observed among food-insecure populations (14). Several studies have documented an inverse relationship between diet quality and food insecurity among United States adults and children (15), with lower-quality diets contributing to worsened glycemic control (16), a higher risk of T2D (17) and other diet-related chronic conditions (18).

However, few studies have assessed relationships between food insecurity among culturally diverse samples including individuals identifying as Hispanic/Latino at high risk of diet-sensitive diseases such as T2D. El Banco por Salud (El Banco), a biobank established in 2017 by the Center for Disparities in Diabetes, Obesity, and Metabolism at the University of Arizona, was created to address health disparities in the Latino community of Southern Arizona (19). El Banco includes primarily self-reported Latino patients (>98%) of Mexican ancestry receiving care at two Federally Qualified Health Centers (FQHCs) in southern Arizona: El Rio Community Health Center and Mariposa Community Health Center. This biobank serves as a repository for comprehensive health data and biospecimens, providing an essential resource for studying diet-sensitive conditions such as T2D in a population at high risk (19).

Previously, a small study examined the association between food insecurity and cardiometabolic health in a sample of urban Mexican immigrants aged 40 to 84 years living on the U.S.-Mexico border (*n* = 75) (20). That study found that 45% of participants were food insecure, and this was associated with both self-reported and clinically diagnosed T2D, though not with other conditions such as hypertension, hyperlipidemia, obesity, or metabolic syndrome (20).

Given the disproportionate burden of obesity and T2D in Latino people of Mexican ancestry, our study aimed (1) to characterize the food insecurity status among Latino individuals of Mexican ancestry of El Banco, and (2) to investigate the impact of food insecurity on cardiometabolic risk factors and glycemic control within this community.

## 2 Materials and methods

### 2.1 Study sample

All participants in this cross-sectional observational study were recruited from El Banco por Salud biobank. With ongoing recruitment since 2017 (19), El Banco includes predominantly participants who self-identify as Latinos, recruited from FQHC partners in southern Arizona (19). Participants included “probands,” who were the original point of contact, and their family members who also agreed to participate. Inclusion criteria for eligible participants were prescreened from their electronic health records for self-reported Latino ethnicity, age 18–75 years, and glycated hemoglobin (HbA1c) of 5.7% or greater. Participants without T2D were family or friends recommended by the initial participants. Demographic and anthropometric parameters were determined as previously described (19). The study was approved by the University of Arizona Institutional Review Board, and all participants signed written informed consent. The REDCap electronic data management system at the University of Arizona was used for HIPAA-compliant data capture, quality assurance, control, and data export (21).

### 2.2 Food insecurity assessment

Food insecurity was evaluated using the 6-item U.S. Household Food Security Module which measures the extent and severity of household food insecurity in the 12 months prior to the data collection (22). Based on the number of affirmative responses to the 6 questions (score 0–6), households were characterized as high/marginal, low, or very low food security. The United States Department of Agriculture generally reports households with high food security or marginal food security as food secure (score 0–1) and households with low food security or very low food security as food insecure (score 2–6) (23). Accordingly, we assessed food security as an either a binary or continuous variable (with higher scores indicating greater food insecurity).

### 2.3 Physical activity assessment

Physical activity was estimated using the Godin-Shephard Leisure-Time Physical Activity Questionnaire (GSLTPAQ) (24). The Leisure Time Score (LTS) was calculated using the equation: LTS = [(number of mild-intensity physical activity sessions per week × 3) + (number of moderate-intensity physical activity sessions per week × 5) + (number of vigorous-intensity physical activity sessions per week × 9)]. Based on the LTS results, physical activity levels were categorized as active (LTS ≥ 24), moderately active (LTS = 18–24), or sedentary (LTS ≤ 18) (24).

### 2.4 Alcohol consumption assessment

A short version of the alcohol use disorders identification test (AUDIT-C) was used to measure alcohol misuse (25). The AUDIT-C is scored on a scale of 0–12 (a score of 0 reflects no alcohol use). Men who presented with scores greater than a score of 4 and women who presented with scores greater than a score of 3 were considered more likely to be harmed from drinking (at risk drinking). The higher the AUDIT-C score, the more likely it is that the patient’s drinking is affecting his/her health and safety (25).

### 2.5 Sociodemographic characteristics

Sociodemographic characteristics were derived from the responses to the questionnaire administered to participants in the El Banco biobank. The characteristics included in this study were: participant type (proband or family member), language spoken at home (Spanish-only, more Spanish than English, both equally, more English than Spanish, or English-only), educational level (less than a high school education or at least a high school diploma), employment status (full-time, part-time, or unemployed), marital status (single, married or in a couple, widowed, divorced, or separated), country of birth (United States or non-United States), type of insurance (Medicare, commercial, Medicaid, none, or unknown), and household income. The mean household income was obtained from zip codes and using the government site.^1^ These characteristics were selected based on their known association with food insecurity, as previously reported (14).

### 2.6 Dietary assessment

Dietary intake was assessed using the Brief Dietary Assessment Tool for Hispanics (26). This screening tool, specifically designed using data from Mexican Americans in the National Health and Nutrition Examination Survey (NHANES III), evaluates fruit, vegetable, and fat intake (servings/day) among Hispanics in community settings. The 23-item Brief Dietary Assessment Tool for Hispanics, developed explicitly for Hispanic/Latino populations, includes two subscales estimating sources of dietary fat and portions of fruits/vegetables consumed per day. Regarding sources of dietary fat, respondents report the frequency of consumption of 16 sources of fat in the past month (e.g., “French fries or fried potatoes”) on a Likert scale ranging from once a month or less to five or more times a week. For fruits and vegetables, respondents indicate the frequency with which they consumed seven types of fruits and vegetables in the past month (e.g., “green salad such as lettuce or spinach”) on a Likert scale ranging from less than once a week to two or more times a day (26). In addition, participants were asked, “How many servings of soft drinks do you consume per day? (One serving corresponds to 1 can or glass)” to assess the daily intake of sugar-sweetened beverages.

### 2.7 Cardiometabolic risk factors

According to the Adult Treatment Panel III, cardiometabolic risk factors were defined as follow: dyslipidemia (triglycerides ≥150 mg/dL), elevated fasting blood glucose (≥5.6 mmol/L), high-density lipoproteins (HDL) cholesterol (<40 mg/dL for men and < 50 mg/dL for women), hypertension (systolic blood pressure > 130 mmHg and diastolic >85 mmHg), large waist circumference (WC) (men ≥40 inch and women ≥35 inch) (27). Each cardiometabolic risk factor was coded as either “1” for having factor or “0” for not having the factor. All measurements were performed by trained study staff in a clinical laboratory setting.

### 2.8 Statistical analysis

Data analysis was conducted using IBM SPSS Statistics software (PASW Version 21.0, SPSS Inc., Chicago, IL, United States). Results are presented as mean ± standard deviation (SD) for continuous variables and as frequency and percentage (*n*, %) for categorical variables. The Kolmogorov–Smirnov test was used to assess the distribution of the data; variables not normally distributed were logarithmically transformed for the analyses. Descriptive statistics were calculated to summarize participant general and sociodemographic characteristics of the entire study population and according to food security status. Differences between food secure and food insecure groups were evaluated using t-tests and Chi-square tests. Linear regression analyses were performed to explore the relationship between food insecurity and HbA1c levels. HbA1c was used as the dependent variable (outcome) and food insecurity as the independent variable (either as a dichotomous or continuous variable). Three models were utilized: Model 1 was unadjusted, Model 2 was adjusted for age, sex, BMI, and WC, and Model 3 included additional adjustments for home language, employment status, marital status, country of birth, insurance status, and household income. Furthermore, a separate linear regression analysis was conducted to investigate the association between dietary components and food insecurity, adjusting for age, sex, BMI, and HbA1c. The assumptions of normality and constant variance were evaluated using histograms of error residuals. Statistical significance was determined for *p*-values <0.05.

## 3 Results

**Figure 1:**
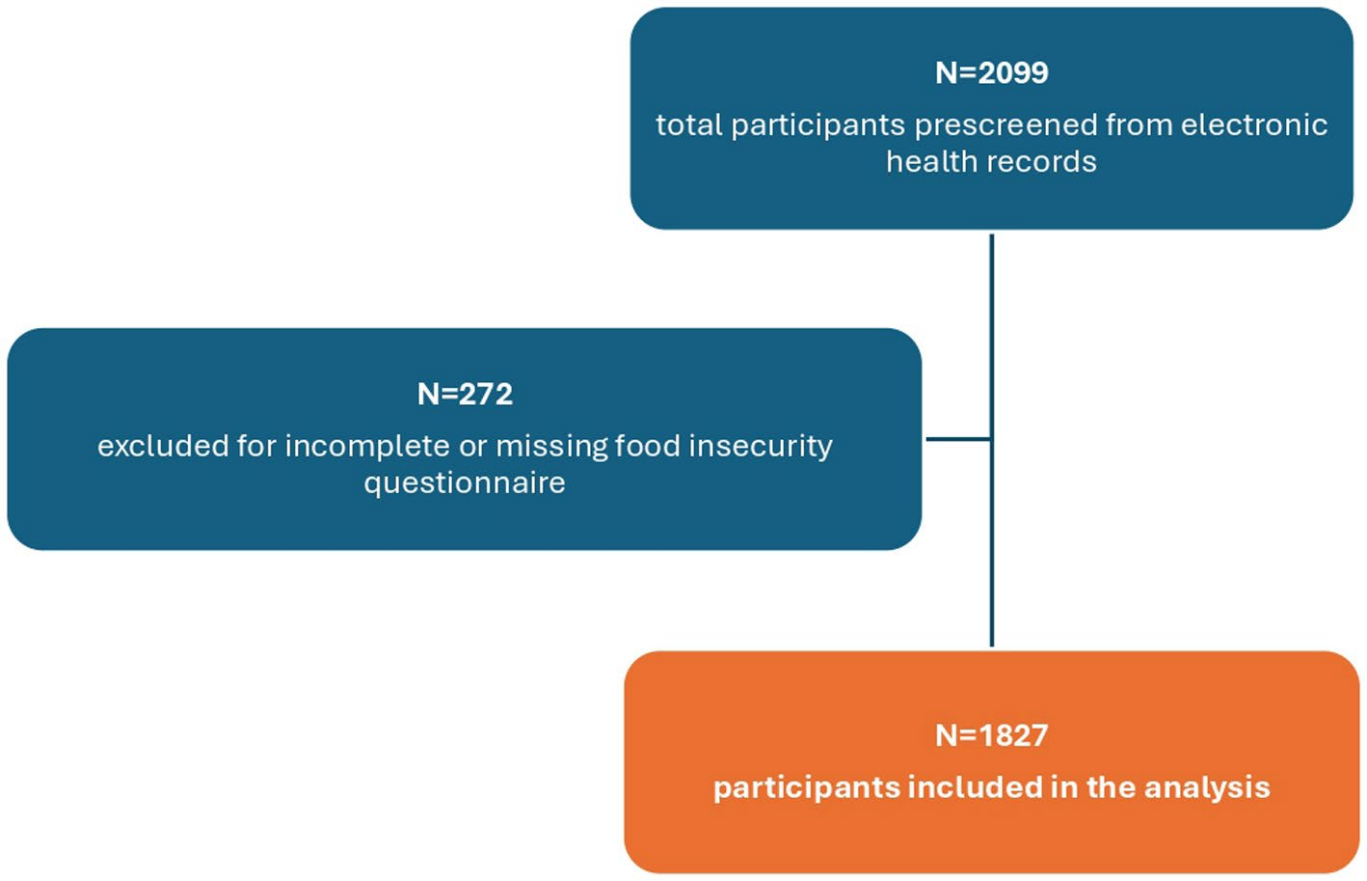
Flowchart of participant recruitment and final inclusion in the study from the El Banco por Salud Biobank.

A flow chart of study participants included in the analyses is shown in Figure 1. Age, sex, BMI, WC, physical activity, and alcohol use of the entire study population and stratified according to food security status are presented in Table 1. The analyses included 1827 participants with a mean age of 52.5 ± 14.4 years. Of these, 1,274 (69.7%) were classified as food secure and 553 (30.3%) as food insecure. Most participants were women (67.6%), were obese (mean BMI 32.4 ± 7.0 kg/m^2^) and were sedentary (43.5%). Food insecure participants had a significantly larger mean WC (*p* = 0.034) compared to food secure participants. When stratified by sex, the difference in WC remained significant in females, with food insecure women having a larger mean WC compared to food secure women (42.1 ± 5.9 vs. 41.1 ± 6.1 inches, respectively, *p* = 0.008), while no significant difference was observed in males. Other results remained unchanged.

**Table 1:** Age, sex, BMI, WC, physical activity, and alcohol use of the entire study population and according to food security status.

Parameters	N of respondents	All	Food secure	Food insecure	*p*
Age (years)	1827	52.5 ± 14.4	52.7 ± 14.6	51.8 ± 14.0	0.345
Sex	1826				
Male		592 (32.4%)	415 (32.6%)	177 (32.0%)	0.786
Female		1,234 (67.6%)	858 (67.4%)	376 (68.0%)	
BMI (kg/m^2^)	1827	32.4 ± 7.0	32.8 ± 7.0	32.7 ± 7.0	0.257
WC (inch)	1819	42.0 ± 6.3	41.8 ± 6.2	42.5 ± 6.3	**0.034**
Physical activity	1807				
Sedentary		786 (43.5%)	560 (44.4%)	226 (41.5%)	0.513
Moderately active		310 (17.2%)	212 (16.8%)	98 (18.0%)	
Active		711 (39.3%)	490 (38.8%)	221 (40.6%)	
Alcohol use	1827				
At-risk drinking		419 (22.9%)	283 (22.2%)	136 (24.6%)	0.266
Not at-risk drinking		1,408 (77.1%)	991 (77.8%)	417 (75.4%)	

BMI, body mass index; WC, waist circumference. The *p* value in bold type denotes a significant difference (*p* < 0.05).

Table 2 presents the sociodemographic characteristics of the entire study population and stratified by food security status. Significant differences were observed in home language, work, marital status, country of birth, insurance coverage, and median household income. Specifically, food insecure participants were more likely to speak English at home (*p* = 0.006), to be single (*p* < 0.001), unemployed (*p* < 0.007) to have Medicare coverage (*p* < 0.001) and less likely to be born in the United States (p < 0.001). They were also more likely to have a lower median household income (*p* = 0.002) compared to food secure participants.

**Table 2:** Sociodemographic characteristics of the entire study population and according to food security status.

Parameters	N of respondents	All	Food secure	Food insecure	*p*
Participant type	1827				
Proband		1,002 (54.8%)	698 (54.8%)	304 (55.0%)	0.942
Family member		825 (45.2%)	576 (45.2%)	249 (45.0%)	
Home language	1799				
Only Spanish		656 (36.0%)	466 (36.7%)	190 (34.4%)	**0.006**
More Spanish than English		414 (22.7%)	309 (24.3%)	105 (19.0%)	
Both equally		319 (17.5%)	222 (17.5%)	97 (17.6%)	
More English than Spanish		292 (16.0%)	187 (14.7%)	105 (19.0%)	
Only English		142 (7.8%)	87 (6.8%)	55 (10.0%)	
Education	1819				
< high school		883 (48.5%)	613 (48.3%)	270 (49.1%)	0.758
≥ high school		936 (51.5%)	656 (51.7%)	280 (50.9%)	
Work	1799				
Full-time		530 (29.5%)	398 (31.7%)	132 (24.3%)	**<0.007**
Part-time		241 (13.4%)	161 (12.8%)	80 (14.7%)	
Unemployed		1,028 (57.1%)	697 (55.5%)	331 (61.0%)	
Marital status	1826				
Single, never married		395 (21.6%)	248 (19.5%)	147 (26.6%)	**<0.001**
Married or domestic partnership		937 (51.3%)	700 (74.7%)	237 (42.9%)	
Widowed		125 (6.8%)	88 (6.9%)	37 (6.7%)	
Divorced		273 (15.0%)	169 (13.3%)	104 (18.8%)	
Separated		96 (5.3%)	68 (5.3%)	28 (5.1%)	
Country of birth	1809				
Not United States		1,087 (60.1%)	795 (63.0%)	292 (53.3%)	**<0.001**
United States		722 (39.9%)	466 (37.0%)	246 (46.7%)	
Insurance	1825				
Medicare		591 (32.3%)	374 (29.4%)	217 (39.2%)	**<0.001**
Commercial		295 (16.2%)	235 (18.5%)	60 (10.8%)	
Medicaid		345 (18.9%)	256 (20.1%)	89 (16.1%)	
None		350 (19.2%)	245 (19.3%)	105 (19.0%)	
Unknown		244 (13.4%)	162 (12.7%)	82 (14.8%)	
Household income	1812	53043.5 ± 12203.3	53633.6 ± 12550.6	51689.6 ± 11261.0	**0.002**

The *p* value in bold type denotes a significant difference (*p* < 0.05).

Table 3 shows the linear regression analyses examining the association between dietary components and food insecurity, adjusted for age, sex, BMI and Hba1c levels. An increase in the food insecurity score (more food insecure) was significantly associated with an increase in the frequency of consumption of hamburgers (*p* < 0.001), pizza (*p* = 0.018), any potatoes (*p* < 0.001) and soft drinks (*p* < 0.001). An increase in the food insecurity score was significantly associated with a reduction in the frequency of consumption of eggs (*p* = 0.004), other mixed dishes with meat (*p* = 0.002), fruit (*p* = 0.003), use fat or oil to fry, cook, or season (*p* < 0.001) and tomatoes/fresh salsa (*p* = 0.003).

**Table 3:** Linear regression analysis with dietary components as dependent variables (outcomes) and food insecurity score as independent variable.

Outcomes	*R*^2^	Beta	CI (95%)	*p*	Overall *p*
Dietary fat sources (times/month)					
Flour tortilla	0.015	−0.037	−0.51 – (−0.05)	NS	<0.001
Refried beans	0.018	−0.025	−0.41 – 0.12	NS	<0.001
Hamburgers/cheeseburgers	0.102	0.092	0.23–0.43	**<0.001**	<0.001
French fried/fried potatoes	0.064	0.037	−0.03 – 0.36	NS	<0.001
Fried chicken	0.009	0.020	−0.10 – 0.27	NS	0.004
Eggs	0.010	−0.067	−0.64 – (−0.12)	**0.004**	0.002
Tacos/burritos/enchiladas	0.035	0.001	−0.21 – 0.22	NS	<0.001
Other mixed dishes with meat	0.045	−0.072	−0.60 – (−0.14)	**0.002**	<0.001
Pizza	0.122	0.053	0.03–0.32	**0.018**	<0.001
Roast pork, beef, or steak	0.025	−0.016	−0.28 – 0.13	NS	<0.001
Cheese/cheese spread	0.005	0.006	−0.45 – 0.06	NS	0.108
Cake, sweet rolls, doughnuts	0.003	0.005	−0.03 – 0.25	NS	0.391
Use fat or oil to fry, cook, or season	0.013	−0.084	−0.80 – (−0.23)	**<0.001**	<0.001
Salad dressing	0.019	0.005	−0.18 – 0.22	NS	<0.001
Potato/corn chips, peanuts	0.063	0.015	−0.15 – 0.50	NS	<0.001
Whole milk	0.008	−0.012	−0.36 – 0.21	NS	0.018
Dietary fruit and vegetables sources (times/week)					
Green salad	0.029	−0.033	−0.76 – 0.12	NS	<0.001
Fresh vegetables	0.016	−0.027	−0.70 – 0.18	NS	<0.001
Fruit juice	0.016	0.018	−0.31 – 0.70	NS	<0.001
Fruit (fresh/frozen/canned)	0.011	−0.71	−1.52 – (−0.32)	**0.003**	0.002
Any potatoes	0.027	0.088	0.26–0.81	**<0.001**	<0.001
Tomatoes/fresh salsa	0.021	−0.051	−1.14 – (−0.06)	**0.030**	<0.001
Vegetable stew/soup	0.005	0.032	−0.11 – 0.57	NS	0.116
Soft drinks (servings/day)	0.058	0.119	0.22–0.49	**<0.001**	<0.001

NS, not significant. The *p* value in bold type denotes a significant difference (*p* < 0.05).

Cardiometabolic risk factors and HbA1c levels of the entire study population and according to food security status are presented in Table 4. Food insecure participants had a significantly higher levels of HbA1c levels (*p* = 0.043) compared to food secure participants.

**Table 4:** Cardiometabolic risk factors and HbA1c of the entire study population and according to food security status.

Parameters	N of respondents	All	Food secure	Food insecure	*p*
Large WC (yes)	1818	1,513 (83.2%)	1,053 (83.0%)	460 (83.6%)	0.756
Dyslipidemia (yes)	1817	827 (45.5%)	569 (44.9%)	258 (47.0%)	0.405
Elevated FPG (yes)	1818	1,135 (62.4%)	784 (61.7%)	351 (64.1%)	0.349
Low HDL (yes)	1781	996 (54.5%)	698 (56.4%)	298 (54.8%)	0.519
Hypertension (yes)	1814	594 (32.7%)	425 (33.6%)	169 (30.8%)	0.255
Hba1c (%)	1827	7.3 ± 2.1	7.3 ± 2.0	7.5 ± 2.3	**0.043**

WC, waist circumference; FPG, fasting plasma glucose; HDL, high density lipoprotein; HbA1c, hemoglobin A1c. The *p* value in bold type denotes a significant difference (*p* < 0.05).

Table 5 presents the results of linear regression analyses examining the association between food insecurity and HbA1c levels, with different models accounting for various adjustments. Food insecurity was significantly associated with higher HbA1c levels in the unadjusted model (*p* = 0.016), and this relationship remained significant after adjusting for age, sex, BMI, and WC (*p* = 0.007). Even with further adjustments for additional sociodemographic factors, the positive association between food insecurity and HbA1c levels continued to be statistically significant (*p* = 0.021).

**Table 5:** Linear regression analysis with HbA1c (%) ad dependent variable (outcome) and food insecurity (score) as independent variable.

Outcome (HbA1c, %)	*R*^2^	Beta	CI (95%)	*p*	Overall *p*
Model 1^a^					
Food insecurity score	0.003	0.056	0.01–0.04	**0.016**	0.016
Model 2^b^					
Food insecurity score	0.087	0.060	0.01–0.34	**0.007**	<0.001
Model 3^c^					
Food insecurity score	0.107	0.053	0.01–0.04	**0.021**	<0.001

^a^unadjusted. ^b^adjusted for age, sex, BMI and WC. ^c^adjusted for age, sex, BMI and WC, home language, work, marital status, country of birth, insurance and household income. The *p* value in bold type denotes a significant difference (*p* < 0.05).

## 4 Discussion

This cross-sectional, observational study analyzed food insecurity in a sample of 1,827 Latino individuals of Mexican ancestry. Approximately 30.3% of participants were classified as food insecure, a higher prevalence than the 20.8% reported in the general Hispanic population according to the most recent USDA report (2022) (3). This higher prevalence is expected given that participants were recruited from a FQHC, which serves underserved and low-income populations. These patients are more likely to experience challenges in availability and access to sufficient and nutritious food. Additionally, previous studies have reported alarmingly high rates of food insecurity among Mexican immigrants and Hispanic farmworkers in the United States (4–7), highlighting the persistent and severe challenges faced by these communities.

Understanding the factors contributing to food insecurity is crucial for addressing its negative impacts on specific populations. Recognizing how various factors intersect and affect food security in vulnerable households is essential for developing or adapting interventions and policies that improve access to and availability of nutritious foods, thereby enhancing health outcomes. In this study, significant disparities were observed between food secure and food insecure participants. Those experiencing food insecurity were more likely to be single, speak only English at home, be unemployed, be born outside the U.S., and have lower household incomes. These findings align with existing literature linking food insecurity to marginalized and socioeconomically disadvantaged populations (28). Additionally, the greater reliance on Medicare among food insecure participants underscores their vulnerability and the need for inclusive social policies. However, the higher proportion of food insecure individuals speaking English at home contrasts with the expected pattern where non-English speakers tend to experience higher food insecurity (28). This discrepancy may reflect variations in acculturation, as English-speaking could indicate different levels of integration and adaptation that do not always align with food insecurity trends. Geographic or demographic variations, sample characteristics, and evolving socioeconomic conditions might also contribute to these differences. Methodological variations in measuring food insecurity could further explain these deviations (28).

Linear regression analyses showed that higher levels of food insecurity were associated with increased consumption of energy-dense, low-nutrient foods like hamburgers, pizza, potatoes, and soft drinks. Conversely, as food insecurity scores increased, the intake of healthier foods such as fruits, eggs, and vegetables decreased. This suggests that food access issues may force individuals experiencing food insecurity to make poorer dietary choices, characterized by high sugar and fat content, which can predispose them to adverse health outcomes. Existing literature supports this association, noting that limited access to nutritious foods often results in unbalanced diets that contribute to negative health impacts, such as poorer glycemic control (16–18).

Racial and ethnic minorities, including those in our study population, are disproportionately affected by the negative health outcomes associated with food insecurity (29). In our study, food insecure participants had significantly higher WC and HbA1c levels compared to their food secure counterparts. Notably, the association between food insecurity and elevated HbA1c levels remained significant even after adjustment for several covariates, indicating that food insecurity, even if in a small part, could play an independent role in contributing to poorer glycemic control. This suggests that the stress and dietary compromises associated with food insecurity may have direct and adverse effects on metabolic health, particularly in populations already at heightened risk for T2D (13). Addressing food insecurity in these communities is crucial not only for improving access to healthier foods but also for mitigating the broader impact on metabolic health and chronic disease management.

The limits of the study are mainly related to the cross-sectional design, which does not allow any conclusion about causality. Some variables, such as dietary intake and sociodemographic factors, were self-reported, introducing the potential for recall bias and social desirability bias, which could affect the accuracy of the findings. While the Brief Dietary Assessment Tool for Hispanics is tailored to the study population, it is not a comprehensive measure of overall diet quality. Consequently, the findings may not fully capture the complexity of dietary patterns or variations in cultural food practices that could influence the relationship between food insecurity and health outcomes. Finally, the study highlighted an unexpected finding: English-speaking participants were more likely to experience food insecurity. This could be due to unmeasured factors related to acculturation or socioeconomic adaptation, which were not fully explored in this analysis.

This study also has several strengths. The study analyzed data from a relatively large sample of 1,827 Latino individuals of Mexican ancestry, providing sufficient statistical power to detect associations between food insecurity and cardiometabolic outcomes. The study specifically targeted Latino individuals of Mexican ancestry, a population that is often underrepresented in research despite being at high risk for food insecurity and related health disparities. We used a valid and reliable instrument to assess food security status, and we considered multiple sociodemographic variables which provided a nuanced understanding of the factors contributing to food insecurity within this population. The inclusion of clinical measures adds robustness to the analysis by providing objective evidence of the impact of food insecurity on metabolic health.

In conclusion, this study has revealed a high prevalence of food insecurity and a significant relationship between food insecurity, diet and glycemic control among Latinos individuals of Mexican ancestry. These findings provide valuable insights for our FQHC partner, El Rio Community Health Center, to address food insecurity and diet disparities among its patients. Future research should explore the feasibility of food-based nutrition interventions, like medically tailored groceries from regional food banks, under the food as medicine framework.

## Data Availability

The raw data supporting the conclusions of this article will be made available by the authors, without undue reservation.

## Footnotes

^1^ census.gov
